# Prioritizing Countries for Interventions to Reduce Child Mortality: Tools for Maximizing the Impact of Mass Drug Administration of Azithromycin

**DOI:** 10.1371/journal.pone.0096658

**Published:** 2014-05-16

**Authors:** Alastair I. Matheson, Lisa E. Manhart, Patricia B. Pavlinac, Arianna R. Means, Adam Akullian, Gillian A. Levine, Julie Jacobson, Erin Shutes, Judd L. Walson

**Affiliations:** 1 University of Washington, Seattle, Washingtion, United States of America; 2 Bill & Melinda Gates Foundation, Seattle, Washington, United States of America; Vanderbilt University, United States of America

## Abstract

**Background:**

As new interventions to reduce childhood mortality are identified, careful consideration must be given to identifying populations that could benefit most from them. Promising reductions in childhood mortality reported in a large cluster randomized trial of mass drug administration (MDA) of azithromycin (AZM) prompted the development of visually compelling, easy-to-use tools that synthesize country-specific data on factors that would influence both potential AZM benefit and MDA implementation success.

**Methodology/Principal Findings:**

We assessed the *opportunity* to reduce mortality and the *feasibility* of implementing such a program, creating *Opportunity* and *Feasibility Indices*, respectively. Countries with high childhood mortality were included. A *Country Ranking Index* combined key variables from the previous two Indices and applied a scoring system to identify high-priority countries. We compared four scenarios with varying weights given to each variable.

Twenty-five countries met inclusion criteria. We created easily visualized tools to display the results of the Opportunity and Feasibility Indices. The Opportunity Index revealed substantial variation in the opportunity for an MDA of AZM program to reduce mortality, even among countries with high overall childhood mortality. The Feasibility Index demonstrated that implementing such a program would be most challenging in the countries that could see greatest benefit. Based on the Country Ranking Index, Equatorial Guinea would benefit the most from the MZA of AZM in three of the four scenarios we tested.

**Conclusions/Significance:**

These visually accessible tools can be adapted or refined to include other metrics deemed important by stakeholders, and provide a quantitative approach to prioritization for intervention implementation. The need to explicitly state metrics and their weighting encourages thoughtful and transparent decision making. The objective and data-driven approach promoted by the three Indices may foster more efficient use of resources.

## Introduction

Although global childhood mortality has declined substantially over the previous 20 years, from 11.6 million deaths in 1990 to 7.2 million in 2011 [1], rates remain unacceptably high in many regions. Infectious diseases, including diarrheal disease, pneumonia, and malaria are responsible for approximately 60% of all deaths in children younger than five years of age [2] and large-scale interventions that target these diseases may have the potential to dramatically reduce childhood mortality. As successful interventions are identified and implemented, systematic approaches for determining which populations would most benefit from these interventions will be essential.

International governmental organizations, development agencies, and non-governmental organizations (NGOs) must decide which interventions to prioritize to reduce childhood mortality and where to conduct the intervention. The methods by which these decisions are made are often opaque. Populations or geographic regions may be targeted based on perceived need, political relationships, existing infrastructure, historical success, or other rationales. In addition, governments, donors, NGOs, and other implementing partners may be subject to unacknowledged external influences such as parallel aid, trade, diplomacy, and national security activities [3]. While important, the selection of areas for implementation based on these factors alone may not result in the most effective or efficient means of reducing disease.

Instead, once interventions have been identified, donor organizations should target the countries or regions that would reap the greatest benefit and in which implementation is most feasible. This requires a more systematic and transparent approach than is commonly practiced.

Promising results from a recent clinical trial of mass drug administration (MDA) of the antibiotic azithromycin (AZM) to control blinding trachoma provided the impetus to develop a data-driven tool for this purpose. The trial resulted in approximately a 50% reduction in mortality among children aged 1–9 years in treated communities compared to control communities (OR: 0.51, 95% confidence interval: 0.29–0.90), even though trachoma is not a direct cause of mortality [4]. Though the widespread adoption of MDA of AZM is controversial given the potential for antibiotic resistance to develop [5], developing a process for prioritizing where to begin implementing such an intervention should take place has wider utility as the techniques can be applied to other fields and interventions.

Our aim was to develop visually compelling, easy-to-use tools that synthesize country-specific data on factors that would influence both potential AZM benefit and MDA implementation success. The tools are designed to assist international decision-makers in accessing and using available data to inform decisions about which countries should be prioritized for MDA of AZM to reduce childhood mortality.

## Methods

We identified countries that could benefit most from an MDA of AZM program by assessing two broad considerations: the *opportunity* to reduce mortality and the *feasibility* of implementing an MDA program. We assumed that countries with high childhood mortality have the greatest opportunity to benefit from MDA of AZM, especially those with a high burden of diseases that are treatable by AZM, such as diarrhea, pneumonia, malaria, and opportunistic infections associated with HIV. We considered countries endemic for trachoma to be of lower priority because the World Health Organization (WHO) currently recommends AZM as part of the S.A.F.E. (surgery, antibiotics, facial cleanliness, and environmental improvement) strategy for eliminating trachoma [6] and children living in areas covered by this strategy may already receive AZM regularly, or will soon be targeted. Similarly, countries with high immunization coverage may expect decreases in childhood mortality in the near future as a result of vaccine protection, which may reduce the impact of an MDA of AZM. Finally, economic stability, violence, corruption, and existing MDA platforms in a given country will influence how feasibly an MDA of AZM program could be implemented. To consider these elements, we created an *Opportunity Index* that summarized factors related to the anticipated benefit of an MDA of AZM program and a *Feasibility Index* that summarized factors related to the ability of a given country to implement a program. Finally, we created a *Country Ranking Index* that combined key variables from the previous two Indices and applied a scoring system to identify high priority countries.

### Country selection

We first identified the twenty countries with the highest estimated childhood (ages 1–4) mortality (deaths per 1,000 live births) globally in 2011, all of which were in Africa [1]. The five countries with the highest childhood mortality from, collectively, the United Nations regions of Central Asia, Southern Asia, South-Eastern Asia, or Oceania were also included to provide wider geographic coverage of high-burden countries.

### Opportunity Index

The Opportunity Index grouped disease- and intervention-related measures expected to influence the potential effectiveness of MDA of AZM into four categories: under-five mortality, trachoma endemicity and programs, disease burden, and vaccine coverage.

*Under-five mortality* was measured by three metrics for each country: (1) the absolute number of deaths among those aged 30 days to four years, inclusive, (2) post-neonatal infant mortality (deaths among those aged 30–365 days per 1,000 live births), and (3) childhood mortality (deaths among those aged 1–4 years per 1,000 live births) [1]. *Trachoma endemicity and programs* was measured by four metrics that captured country-level trachoma burden and present or future trachoma-specific AZM programs: (1) trachoma endemicity in 2012 [7], (2) the proportion of the population living in non-endemic areas [7]–[9], (3) coverage of existing AZM programs [10], and (4) the target date for elimination of blinding trachoma [11], [12]. *Disease burden* was measured by four metrics summarizing mortality from AZM-treatable or AZM-preventable diseases (diarrhea [13], pneumonia [14], malaria [13], and HIV [13]; for HIV this referred to co-infections). Finally, we included *vaccine coverage* to capture the potential future reduction in childhood mortality from vaccine-preventable diseases, which would attenuate the population-level impact of AZM. This set of metrics included coverage rates for measles; diphtheria, tetanus, and pertussis (DTP); polio; and *Haemophilus influenzae* type B (Hib) vaccines [15], and the planned or actual introduction date for rotavirus and pneumococcal vaccines [16]–[19].

For each metric, countries were categorized into high-, medium- or low-opportunity groups and color-coded as blue, yellow and red, respectively, for rapid visualization of high-opportunity countries. Where possible, the cutoff points for each category were defined as the tertile values in the 50 highest-burden countries for that condition. We found that 50 countries sufficiently allowed for differentiating between the countries of interest (using more countries to create tertiles would place most countries of interest into the high-opportunity category and using too few countries would place most countries of interest into the low-opportunity category). When this approach was not feasible due to insufficient breadth of data (all trachoma measures except program coverage) or there was a stronger rationale for absolute cutoff points (vaccine coverage and implementation dates), we determined cutoff points following group discussion of where meaningful boundaries likely exist. For the trachoma program coverage metric, the percent of the ultimate intervention goal for antibiotics achieved (UIG-A%), we divided the countries in the Index into tertiles because of insufficient data from other countries. Cells for which no data were available were left blank. Operational definitions and cutoff points for the measures are summarized in Table S1.

### Feasibility Index

We considered the following as important indicators of the feasibility of operating a large-scale health intervention: economic stability, presence of violent conflict, corruption, government expenditure on health, and existing MDA platforms. Economic stability and violent conflict are indicative of the risks involved in investing resources in a country. Corruption increases costs and can lengthen the time it takes to implement a program. The percent of government expenditure devoted to health served as a proxy for how a country prioritizes health. Finally, existing MDA programs represented health infrastructure that could be leveraged, potentially reducing the cost and time needed to begin operating an MDA of AZM program. We measured economic stability as the grade on the Euler Hermes Risk Rating System [20], presence of violent conflict from the Global Peace Index [21], corruption by score on the 2013 Transparency International Corruption Perceptions Index [22], expenditure on health as a percent of total government expenditure from WHO estimates [23], and existing MDA platforms by presence of an MDA program for lymphatic filariasis, soil-transmitted helminths, schistosomiasis or malaria in pregnancy [7], [24].

Similar to the Opportunity Index, we placed countries into categories of high, medium, and low feasibility and used the same color-coding scheme described above. The cutoff points for all variables except the Global Peace Index (GPI) were determined after discussion and group consensus was achieved, as no universally accepted ranking systems or cut off criteria for these characteristics exist. For the Global Peace Index we defined tertiles from the scores of the 50 highest-scoring countries. Operational definitions and cutoff points for the measures are summarized in Table S1.

### Country Ranking Index

The Country Ranking Index combined the most relevant data from the Opportunity and Feasibility Indices to prioritize countries for implementing an MDA of AZM: the proportion of the population living in areas not endemic for trachoma (taken directly from the Opportunity Index) and four weighted composite scores for under-five mortality, disease burden, immunization coverage, and feasibility (compiled from individual categories in the Opportunity and Feasibility Indices).

To create the composite scores, we assigned numeric values to each category of opportunity or feasibility in those Indices. High opportunity/feasibility was scored as 3, medium as 2, and low as 1. For the one instance of missing data in government expenditure on health, we took an average of that country's scores in the other feasibility variables. These numeric values were weighted and summed to produce a single score for each of the mortality, disease, vaccine coverage, and feasibility composite measures. The weighting schemes accounted for differences in the relative importance of each individual measure (Table S1). Briefly, the mortality and disease burden weighting schemes gave equal weight to each measure, the immunization coverage scheme gave greater weight to vaccines targeted at diseases potentially impacted by AZM, and the feasibility composite measure applied greater weight to measures most likely to hinder or facilitate an MDA of AZM, namely violence, corruption, and the number of existing MDA platforms.

We then divided metrics within the Country Ranking Index into high, medium and low priority categories, as described in Table S1. For the four composite variables, we divided the countries into tertiles. We calculated a crude c*ountry score* for each country by using the same scoring system of 3 points for high, 2 for medium, and 1 for low rankings and summing the total of all variables. In addition, we calculated a w*eighted country score* that reduced the weight of the immunization coverage and feasibility composite scores. We reasoned that, while immunization programs would likely reduce future childhood mortality and decrease the effect of an MDA of AZM program, there may be other causes of morbidity and mortality that could be impacted by an MDA of AZM program. In the case of feasibility, we expected that accommodations could be made during program design to surmount the challenges of conducting such a program. However, in order to assess the effect of these assumptions, we conducted a sensitivity analysis with two additional weighting schemes: 1) doubling the weight of the feasibility score and weighting all other scores at one, and 2) giving trachoma a half weighting and weighting all other scores at one. We then compared the five highest-scoring and five lowest-scoring countries under each scenario.

## Results

Twenty-five countries met our criteria for inclusion and are summarized in the Indices. The childhood mortality rate ranged from 15 deaths per 1,000 live births in Myanmar to 92per 1,000 live births in Niger, with a median of 49.5 per 1,000 live births. Collectively, the 25 countries had an estimated 2,069,900 deaths among children aged 30 days to 4 years in 2011, 63.4% of which occurred in just five countries: Nigeria, the Democratic Republic of Congo, Pakistan, Afghanistan, and Niger (Table S2).

### Opportunity Index

Approximately three-quarters of the countries had high opportunity in terms of potential for reduction in post-neonatal and childhood mortality rates, which was expected given that the latter was a criterion for country selection (Table S2). However, 10 of the 16 countries with highest childhood mortality rates were in the medium- and low-opportunity categories in terms of the absolute number of childhood deaths. Although 21 of the 25 countries were endemic for trachoma, 6 of these had a high proportion of the population living in areas where trachoma was not endemic, indicating high opportunity. In addition, 8 endemic countries had either no trachoma program or low trachoma program coverage, indicating that some countries where trachoma is endemic may still have high opportunity for an MDA of AZM. Opportunity was not consistently high in the burden of disease metrics, with only 3 countries having a high opportunity level across all four diseases (Cameroon, Chad, and Nigeria), and 9 countries having high opportunity in three diseases. Coverage rates for immunizations were generally medium to high, indicating low to moderate opportunity.

### Feasibility Index

Countries varied substantially in the metrics associated with the feasibility of implementing a successful MDA of AZM program (Table S3). Most (18 of 25) of the countries received the lowest possible grade for economic stability, implying some risk for any intervention. A majority of countries were in the highest tertile derived from the GPI, but there was significant variation in scores. The country with the lowest GPI score (Lao PDR), indicating a more peaceful country, was ranked 39th out of all nations whereas the country with the highest GPI score (Afghanistan) was ranked the least peaceful country in the world. Most (20 of 25) countries listed fell in the bottom third of possible transparency scores, indicating potential challenges to implementing and operating a program. Similarly, more than half (14 of the 25 countries) spent less than 10% of total government expenditure on healthcare in 2011, which may reflect lower national prioritization of health and greater attention to other sectors or expenditures. Finally, all countries except Pakistan had at least one MDA platform already in place and 15 had three or four platforms, suggesting that there was experience with MDA programs that could be leveraged to support an AZM MDA program.

### Country Ranking Index

**Figure 1:**
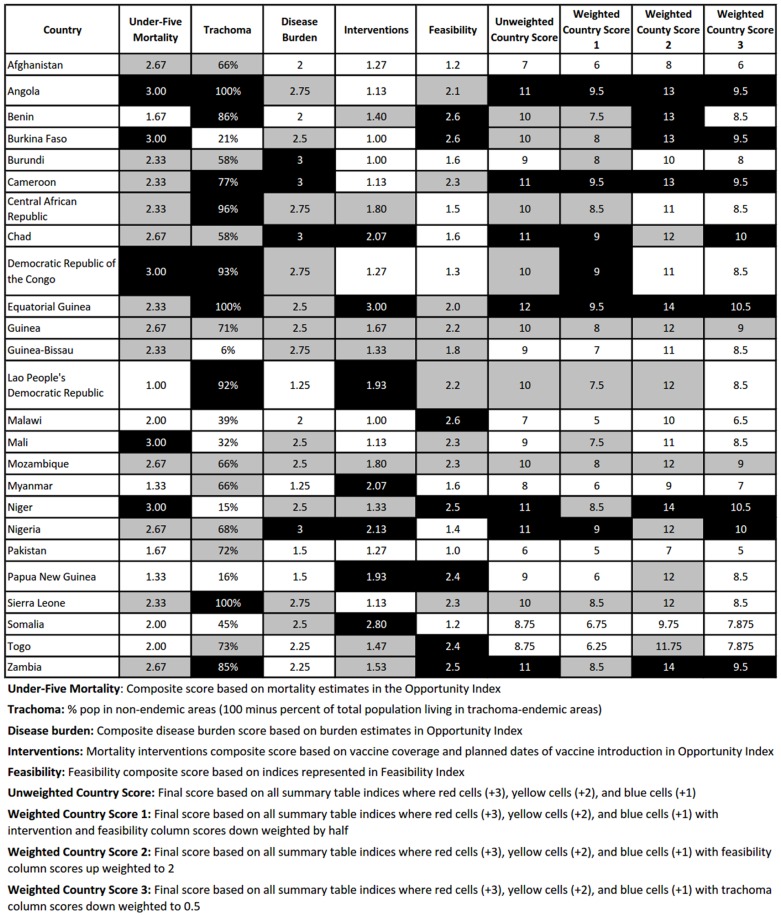
The Country Ranking Index summarizes opportunity and feasibility for implementing an azithromycin mass drug administration (darkest  =  highest ranked tertile, lightest  =  lowest ranked tertile).

The unweighted country scores ranged from a low of 6 (Pakistan) to a high of 12 (Equatorial Guinea) (Figure 1, see Figure S1 for the color version). Once weighting was applied, scores ranged from 5 (Malawi and Pakistan) to 9.5 (Angola, Cameroon, and Equatorial Guinea). In sensitivity analyses, the groups of countries with the highest and lowest scores remained fairly consistent when different weighting schemes were employed (Table 1).

**Table 1:** Highest- and lowest-ranked countries under different weighting scenarios.

	Rank	Unweighted	Scenario 1	Scenario 2	Scenario 3
Highest scores	1	Equatorial Guinea	Angola	Equatorial Guinea	Equatorial Guinea
	2	(2 = ) Angola, Cameroon, Chad, Niger, Nigeria, Zambia	Cameroon	Niger	Niger
	3		Equatorial Guinea	Zambia	Chad
	4		(4 = ) Chad, DRC, Nigeria	(4 = ) Angola, Benin, Burkina Faso, Cameroon	Nigeria
	5				(5 = ) Angola, Burkina Faso, Cameroon, Zambia
Lowest scores	21	(20 = ) Somalia, Togo	(21 = ) Afghanistan, Myanmar, PNG	(20 = ) Burundi, Malawi	(20 = ) Somalia, Togo
	22	Myanmar		Somalia	Myanmar
	23	Afghanistan		Myanmar	Malawi
	24	Malawi	Malawi	Afghanistan	Afghanistan
	25	Pakistan	Pakistan	Pakistan	Pakistan

Scenario 1: Immunization and feasibility scores weighted to 0.5, other scores weighted at 1.

Scenario 2: Feasibility score weighted to 2, other scores weighted at 1.

Scenario 3: Trachoma score weighted to 0.5, other scores weighted at 1.

DRC  =  Democratic Republic of the Congo.

PNG  =  Papua New Guinea.

## Discussion

This series of tools can assist donor organizations in identifying countries most likely to successfully reduce childhood mortality through an MDA of AZM program. They can also be adapted for use with a variety of other interventions. While the Opportunity Index in particular is targeted at an AZM intervention, the general framework has wider applicability to other programs. Many countries may benefit from interventions to reduce childhood mortality and the tools provide a useful mechanism to prioritize countries for interventions using a data-driven, systematic assessment of opportunity and feasibility.

The Opportunity Index revealed that even among countries with high overall childhood mortality, there was substantial variation in the opportunity for an MDA of AZM program to reduce mortality. The variation was especially noticeable in the disease burden metrics, where countries tended to fall into the high opportunity category in two or three diseases, but low or medium opportunity categories in others. Countries that had high opportunity in terms of disease burden sometimes had low opportunity in terms of vaccine coverage. This finding supports the approach of including a wide range of metrics when using the Index to target implementation of interventions such as MDA of AZM, where the mechanism of action is broad or unknown.

The Feasibility Index demonstrated that implementing a large-scale MDA of AZM would be challenging in many of the countries that could benefit most from such a program. Corruption and economic stability were issues in almost all the countries examined, and it was primarily the presence or absence of violence and existing MDA platforms that accounted for variance in the country scores. The Feasibility Index could be enhanced by gathering evidence on the particular aspects of corruption and economic stability that have a larger impact than others on the success of MDA programs, which would enable finer distinctions to be made in these two indicators.

The Indices have several strengths. They are data driven, which enables empiric comparisons among countries and informs decision making processes. Information is condensed into a visually accessible format; the color coding enables users to tell at a glance the opportunity, feasibility or overall potential benefit of an MDA of AZM in a particular country, and compare relative differences between countries. There is also substantial flexibility in the weighting and selection criteria, which allows for adaptation as new data become available and for decision makers to prioritize different factors based on their relevance to specific questions or contexts. In the case of our application of the Indices to an MDA of AZM, the four weighting scenarios we assessed yielded similar results, indicating that our choice of metrics was fairly robust. The need to explicitly state these prioritizations encourages transparent decision making.

There are also limitations to the tools. They are not designed to inform intervention selection, but instead are intended for use once an intervention has been identified. The very process of selecting a particular intervention from alternatives is often subject to political influence. It may be possible to adapt the tools to allow comparison of different interventions and selection of the optimal one for a particular country but other tools already exist for this purpose, such as the Lives Saved Tool (LiST) [25]. Instead, the Indices could be enhanced by including consideration of existing or planned interventions in a particular country (beyond immunization) and prioritizing high-need countries that are not likely to already be receiving interventions. This is also not the first tool developed for ranking countries in terms of feasibility of achieving a health goal [26].

To compare a large number of countries, we required standardized metrics that are widely collected. However, these standardized data may not always reflect the most accurate or appropriate measure of disease burden, intervention coverage or feasibility. The quality of the data also likely varies across countries. In order for these types of tools to be accurate and useful, countries, intergovernmental entities and organizations need to coordinate and standardize data collection and data sharing efforts. There may be additional factors or activities not identified that could interfere with the potential opportunity or feasibility within a country, for example, the willingness of that country's government to introduce an MDA of AZM program. We did not include a cost-effectiveness metric and this is a critical consideration in determining where to deploy an intervention. Including a cost-effectiveness parameter in the Feasibility Index may enhance the utility of these tools in future adaptations. Data were analyzed at the national level, which may mask heterogeneity within that country. Although we attempted to use an evidence-based approach to identifying cutoff points, the paucity of published data often required subjective decisions. The selection of countries for inclusion in the Indices was an arbitrary decision, made based on areas that traditionally receive the most development aid and a desire to add geographic diversity. Other African countries not in the top 20 for childhood mortality may have greater opportunity and feasibility for an MDA of AZM than the 5 countries selected from Asia and Oceania, and countries in other regions could also likely benefit from such an intervention. Areas of subjectivity, including which countries to include in an analysis, remain a challenge to empirical decision making. The rationale behind, and limitations of, these decisions should be clearly documented when using the tools to foster transparency and encourage debate.

Development assistance for health (DAH) has undergone a huge expansion in funding over the past decade, growing from approximately US$9.86 billion (bn) in 2000 to US$28.1bn in 2012 (both in 2010 US$), though this has recently plateaued with the recent global recession [27]. Despite these increases, funds are not necessarily allocated to countries or communities with the greatest need. Researchers have noted that the distribution of global health financing is misaligned in terms of the burden of disease, the size of the population at risk, or the ability to prevent new infections [28]–[30]. As resources for effective interventions become available, the means by which they are allocated among countries is subject to several factors such as country income levels and disease burden, and a country's ability or willingness to contribute funds [31]. However, the relative importance of such factors may be unclear, leading to uncertainty about why some countries are prioritized over others. The three Indices developed here provide a means to transparently and objectively apply criteria to this process.

Although we developed the three Indices to inform the specific question of where to focus a potential AZM MDA program, the approach could readily be adapted for other interventions or refined to include additional metrics. Our method is particularly suited to complex scenarios where several factors influence the success and impact of a program, or where interventions will likely impact a range of diseases or health outcomes. The objective approach promoted by the three Indices can lead to more effective programs and more efficient use of resources, which should ultimately result in improved health outcomes.

## Supporting Information

Figure S1 **The Country Ranking Index summarizes opportunity and feasibility for implementing an azithromycin mass drug administration (blue  =  highest ranked tertile, red  =  lowest ranked tertile).**
(XLSX)

Table S1 **Metrics and cutoff points used in each Index.**
(XLSX)

Table S2 **The Opportunity Index summarizes the potential for reducing under-five mortality through mass distribution of azithromycin.**
(XLSX)

Table S3 The Feasibility Index summarizes the practical implications of conducting a mass distribution campaign.
(XLSX)
